# Permanent Neonatal Diabetes Mellitus: Same Mutation, Different Glycemic Control with Sulfonylurea Therapy on Long-Term Follow-up

**DOI:** 10.4274/Jcrpe.524

**Published:** 2012-06-09

**Authors:** Banu Küçükemre Ay, Rüveyde Bundak, Firdevs Baş, Hülya Maraş, Nurçin Saka, Hülya Günöz, Feyza Darendeliler

**Affiliations:** 1 İstanbul University Faculty of Medicine, Department of Pediatric Endocrinology, İstanbul, Turkey; +90 212 414 20 00 bkucukemre@yahoo.com

**Keywords:** diabetes mellitus, ABCC8, SUR1, sulfonylurea

## Abstract

Permanent neonatal diabetes mellitus (PNDM) is a rare condition presenting before six months of age. Mutations in the genes encoding the ATP-sensitive potassium (KATP) channel are the most common causes. Sulfonylurea (SU) therapy leads to dramatic improvement in diabetes control and quality of life in most patients who carry these mutations. Here, we report the long-term follow-up results of two siblings with PNDM who were treated with insulin until ABCC8 gene mutation was identified, and were successfully transferred to oral SU therapy. After 3.5 years of follow-up on SU, one patient had a very good response, while the other one had a poor response. Bad compliance to diet was thought to be the most probable reason for poor glycemic control in this patient. In conclusion, molecular genetic diagnosis in all patients with PNDM is recommended. Compliance to treatment should be an important aspect of the follow-up of these patients.

**Conflict of interest:**None declared.

## INTRODUCTION

Permanent neonatal diabetes mellitus (PNDM) is a form of insulin-requiring diabetes presenting before six months of age and is likely to be non-autoimmune in nature. It is a rare condition occurring in only 1.43-1.96/ 100 000 infants (1). Affected infants frequently present with symptomatic hyperglycemia and sometimes with ketoacidosis (2). As a result of lower foetal insulin production, birth weight is low in most infants with PNDM (1). It is now accepted that most neonates and infants presenting with diabetes within the first 6 months of life have a monogenic form of disease although the responsible gene remains unknown in up to 40% of patients (3). The most common causes of PNDM are mutations in the genes (KCNJ11 and ABCC8) encoding the two protein subunits [Kir6.2 and sulfonylurea receptor 1 (SUR1), respectively] of the ATP-sensitive potassium (KATP) channel and in the gene encoding insulin itself (3,4,5,6,7,8). KATP channel is a critical regulator of beta-cell insulin secretion. Insulin secretion is initiated by closure of the channels and inhibited by their opening. The KATP channel is an octameric complex consisting of four Kir6.2 and four SUR1 subunits. In case of activating mutations in Kir6.2 or SUR1, the KATP channel remains open leading to impaired insulin secretion and neonatal diabetes. In contrast, loss-of-function mutations in SUR1 or Kir6.2 lead to congenital hyperinsulinemia by the same mechanism (9). Identification of the underlying genetic cause has led to improved treatment for patients with a mutation in KCNJ11 or ABCC8. These patients usually respond to high-dose sulfonylurea (SU) therapy, with significantly improved glycemic control (10,11).In this paper, we report the long-term follow-up of two siblings with PNDM who were treated with insulin until ABCC8 gene mutation was detected, and who were transferred from insulin to SU. 

## CASE REPORT

**Patient 1**: The first patient was a male infant, diagnosed with diabetes at the age of 5 months in another hospital. According to this hospital’s report, his physical examination was normal when he first presented with focal seizures. His routine laboratory analyses revealed normal serum chemistry, except for high blood glucose levels (528 mg/dL), and normal values for blood gases. Insulin therapy was started and the patient stayed for one month in that hospital. One month later, the infant was referred to our hospital for glycemic regulation; stable metabolic control was achieved with 0.5 U/kg/day NPH insulin. The patient’s initial HbA1c and insulin levels were 12.5% and 5.8 uU/mL, respectively; exocrine pancreas functions were normal. Examination of the stool for occult blood, fat, meat fibers and pH revealed no pathology. His cranial imaging and EEG were unremarkable. The seizures did not recur, and his neuromotor development was normal during the follow-up.

**Patient 2:** The second patient was a 2.5-month-old male infant whose blood glucose was checked because of a history of PNDM in his older brother (Patient 1, presented above). The infant was admitted to our hospital with a blood glucose level of 570 mg/dL. The parents stated that they had not observed any symptoms and reported a weight gain of 2 kg in the first 2 months of life. Physical examination, venous blood gas and electrolyte levels were all normal. HbA1c level was 8.9%. The patient was discharged with 0.4 U/kg/day insulin therapy.

**Figure 1:**
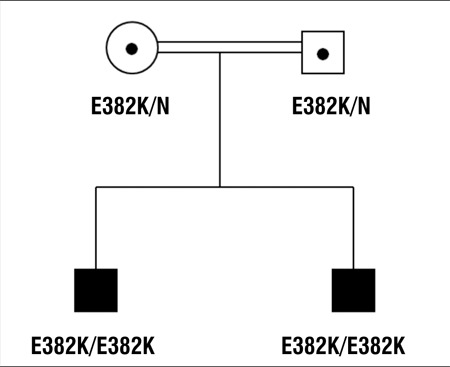
Pedigree of the family

Both patients were followed and received insulin treatment until they were 15 2/12 and 10 9/12 years old, at which time their diagnosis of diabetes was established to be due to an ABCC8 gene mutation, identified by sequencing analysis in Exeter, U.K. Genetic studies revealed a novel homozygous missense mutation, p.E382K, in exon 7 of ABCC8 gene (12). This G>A mutation at nucleotide 1144 (c.1144G>A) results in the substitution of lysine (basic charged polar amino acid) for glutamic acid (acidic charged polar amino acid) at codon 382 (p.Glu382Lys). The glutamic acid residue at codon 382 is conserved across species. This result was consistent with the diagnosis of recessively inherited neonatal diabetes due to the mutation in the SUR1 subunit of the KATP channel. The parents were first-degree cousins and both of them were heterozygous for the E382K missense mutation (Figure 1).

The patients were hospitalized in our clinic for a change of their therapy from insulin to SU. Prior to SU treatment, total daily insulin dose was 0.9 U/kg/day in the older patient and 1.0 U/kg/day in his younger brother. Their HbA1c levels were 8.1% and 8%, respectively. Oral glibenclamide was started in a dose of 0.1 mg/kg/day and gradually increased up to a final dose of 0.8 mg/kg/day twice daily in one week. Insulin requirements were decreased by 50% within one week. At the end of the week, C-peptide levels increased from 0.07 to 2.2 ng/mL and from 0.17 to 2 ng/mL, respectively. Insulin therapy was stopped at the end of two months. Blood glucose levels remained within normal ranges on glibenclamide therapy. HbA1c levels decreased to 6.3% and 6.4% (normal ranges 4.5-6.5%) in patient 1 and 2, respectively (Table 1).

Currently, the patients have been on SU therapy for 3.5 years, and no side effects were reported. The older sibling has a good glycemic control (HbA1c 6.7%) with a lower dose of SU (0.4 mg/kg/day), but the younger has poor glycemic control (HbA1c 10.1% three months prior to the last visit and 7.9% at the last visit) even with a higher dose of SU (0.6 mg/kg/day) (13). Both patients have low body mass indices (BMI) 16.7 kg/m2 [-2.86 standard deviation score (SDS)] and 17.5 kg/m2(-1.45 SDS), respectively. With glucagon stimulation, C-peptide level was higher in the younger sibling (1.64 vs. 2.24 ng/mL) (Table 1). The younger sibling claimed he was well compliant with the SU treatment, but he admitted not being completely compliant with his diet. The parents confirmed that their son was regularly taking the SU tablets. Before increasing the SU dose, we decided to follow the patient’s compliance to the current therapy. Over the following 3 months, the patient showed an increasingly good compliance to the diet and his blood glucose control significantly improved without any increase in SU daily dose and the HbA1c level decreased below 8%. 

## DISCUSSION

The discovery of a genetic background in PNDM had a large impact on the management of the affected subjects. Most of the patients with the molecular diagnosis of PNDM resulting from KCNJ11 and ABCC8 gene mutations could be transferred from insulin to SU treatment. SU are drugs of choice to treat type 2 diabetes mellitus, they close KATP channels by an ATP-independent route, thereby causing insulin secretion. They can close these channels even when mutations are present, so SU are a better treatment option in PNDM caused by mutations in these channels (5,6,10,11).

Due to the mutated KATP channels in neural tissue, some PNDM patients exhibit developmental delay, epilepsy and neonatal diabetes (DEND) syndrome. Especially in patients with a milder form, referred to as the intermediate DEND syndrome, even neurological symptoms can improve with SU therapy (14). Since our patients did not have developmental delay, epilepsy or muscle weakness, a diagnosis of DEND syndrome was ruled out.In PNDM caused by mutations in KATP channels, glycemic control with SU is often better than that accomplished by insulin, as also observed in our patients at the beginning of their therapy. After 3.5 years of follow-up, although both siblings carried the same ABCC8 gene mutation, they showed a different glycemic control on SU.

Unsuccessful switching from insulin to SU has been reported in 10% of children and adults who are KATP channel mutation carriers (15). The failure of SU in some of these patients can be, at least partially, explained by the severity of their alterations. Nevertheless, additional factors, such as the age of the patient, can influence this response (1). Sometimes, even identical mutations within the same families can produce a variable clinical picture (15,16,17). However, no case of secondary failure of SU in PNDM has been reported so far (1). About 50% of type 2 diabetic patients treated with SU experience a severe deterioration of metabolic control within 6 years of therapy initiation. This phenomenon may derive from a loss of insulin secretory capascity due to the hyperexcitability of pancreatic beta cells chronically exposed to this drug (18). Lafusco et al (18) investigated 11 patients who were carriers of KATP channel mutation and had been on SU therapy for more than 57 months and showed that chronic SU therapy retains its efficacy for a long period of time in patients with PNDM, contrary to findings reported in type 2 diabetes.

Compliance is one of the most important practical aspects in long-term treatment, especially in adolescents (19). In our patients, the poor glycemic control of the younger, adolescent sibling was not due to SU secondary failure. With good compliance to his diet and medication, his blood glucose control significantly improved without any increase in SU dose. Although the patient and his family reported good compliance with SU treatment, it could be argued that the HbA1c increase might be due to the skipping of SU tablets rather than just dietary noncompliance. The low BMI of this patient also supports this argument.In conclusion, molecular genetic diagnosis is recommended in all patients with PNDM since the identification of Kir6.2 or SUR1 mutations allows a successful change to SU therapy that leads to dramatic improvement in diabetes control and quality of life. However, these patients should be followed closely for compliance to treatment, especially when they are adolescents.

**Acknowledgments**

The authors are grateful to Andrew Hattersley and Sian Ellard (Peninsula Medical School, Exeter, United Kingdom) for performing the genetic testing of the patients.

## Figures and Tables

**Table 1:**
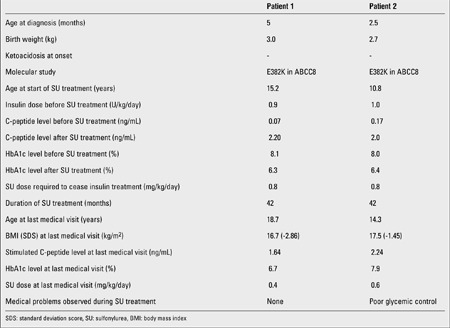
Clinical characteristics of patients according to success of SU treatment
